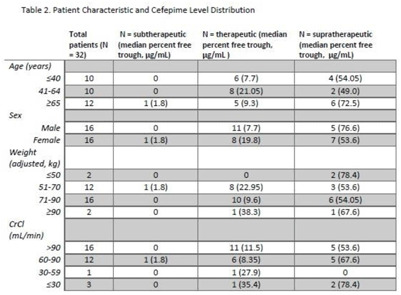# Assessing the association between cefepime percentage free trough level and neurotoxicity

**DOI:** 10.1017/ash.2022.100

**Published:** 2022-05-16

**Authors:** Aleena Zia, Armisha Desai, Molly Tieman, Haley Pritchard

## Abstract

**Background:** Cefepime has a known association with neurotoxicity due to its ability to cross the blood–brain barrier. The symptoms of neurotoxicity are highly variable. It has been postulated that cefepime neurotoxicity is associated with elevated levels of the drug. However, studies assessing for an association between serum drug level and the incidence of neurotoxicity have yet to establish a consistent threshold. We assessed serum cefepime levels and incidence of neurotoxicity to help develop a dosing strategy to minimize adverse effects. **Method:** In total, 32 inpatients admitted from January 2019 to November 2021 who received cefepime according to institutional standard dosing regimens for at least 72 hours were reviewed by infectious diseases pharmacists who obtained serum cefepime levels and performed pharmacokinetic analyses to obtain percentage free trough levels. Cefepime percentage free trough levels were defined as therapeutic if they were above the known minimum inhibitory concentration (MIC) of the treated organism and were <40 μg/mL. Patient charts were reviewed for clinical findings consistent with cefepime-induced neurotoxicity. Numerical and statistical analyses were performed to assess factors with a significant association with neurotoxicity. **Results:** Overall, 16 (47.1%) patients showed some evidence of neurotoxicity, 9 (56.3%) of whom had a likely alternate clinical cause of symptoms (Table 1). We did observe that patients with creatinine clearance <60 mL/min were more likely to have symptoms concerning for neurotoxicity. **Conclusions:** Cefepime percentage free trough levels were highly variable, and no association with neurotoxicity was observed. Patients with decreased creatinine clearance were significantly more likely to develop neurologic findings consistent with cefepime-induced neurotoxicity. Further study is needed to establish a relationship between cefepime pharmacokinetic values and incidence of neurotoxicity.

**Funding:** None

**Disclosures:** None

**Table:**
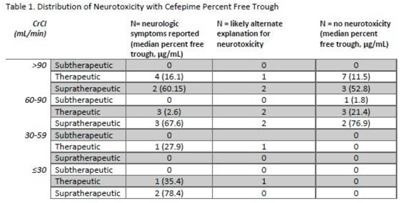

**Table:**